# Distal Esophageal Spasm: An Updated Review

**DOI:** 10.7759/cureus.41504

**Published:** 2023-07-07

**Authors:** Eli A Zaher, Parth Patel, George Atia, Surendra Sigdel

**Affiliations:** 1 Internal Medicine, Ascension Health - Saint Joseph Hospital, Chicago, USA; 2 Gastroenterology and Hepatology, Ascension Health - Saint Joseph Hospital, Chicago, USA

**Keywords:** nitric oxide (no), peroral endoscopic myotomy (poem), dysphagia, chicago classification, functional lumen imaging probe, high resolution manometry, distal esophageal spasm

## Abstract

Distal esophageal spasm is characterized by premature contractions of the distal esophageal smooth muscle leading to non-obstructive dysphagia and non-cardiac chest pain. Diagnosis requires the presence of symptoms along with evidence of at least 20% premature contractions in the setting of a normal lower esophageal sphincter relaxation on high-resolution manometry. New updates to the Chicago Classification have improved the diagnostic accuracy of this method. Functional lumen imaging probe is a growing diagnostic modality that gives a more complete picture of esophageal motility. Pharmacologic treatment remains inadequate. Endoscopic myotomy might be of benefit for non-achalasia esophageal motility disorders. More research is required to better understand the pathophysiology and develop safe and long-lasting management for this disease.

## Introduction and background

Distal esophageal spasm (DES) is an idiopathic motility disorder of the esophagus that can cause significant discomfort and impairment of quality of life in affected individuals. The condition is characterized by abnormal smooth muscle contractions in the distal esophagus, leading to symptoms such as dysphagia, chest pain, and regurgitation. DES is a rare condition that can be difficult to treat, partly due to the limited understanding of its pathophysiology. The diagnosis of DES has traditionally relied on subjective assessments of symptoms and radiographic imaging. However, the invention of high-resolution manometry (HRM) in 2000 and the functional lumen imaging probe (FLIP) in 2003 has enabled a more accurate and objective evaluation of esophageal motility disorders, including DES. HRM is the current gold standard for diagnosis and is interpreted using the Chicago Classification (CC) [1]. This review paper aims to provide a comprehensive overview of the current state of knowledge on DES, including its clinical presentation, pathophysiology, diagnostic workup, and management while incorporating the recent updates to the CC and benefits of FLIP.

## Review

Epidemiology

The prevalence of DES is not precisely known as it is a rare condition. Its reported prevalence varies depending on the population studied as well as the diagnostic criteria used. Additionally, DES was referred to as diffuse esophageal spasm until the recent introduction of the CC in 2008, when it was re-classified as a separate diagnosis. Studies have estimated that the prevalence of DES is around 2% to 9% in symptomatic patients undergoing esophageal motility testing. It is more common in women with a median age of 60 at diagnosis [2-4].

Pathophysiology

DES involves abnormal coordination within the smooth muscles of the esophagus, likely stemming from an imbalance between the nitrogenic inhibitory and cholinergic excitatory pathways. Normally, there is a gradual gradient of inhibitory signals observed along the esophagus, spanning from the proximal to distal regions. As the neuronal signal advances towards the distal esophagus, this inhibitory gradient intensifies. Consequently, the duration of deglutitive inhibition progressively elongates as the peristaltic wave traverses toward the distal segments of the esophagus. This specific period of deglutitive inhibition, referred to as contractile latency, has been recognized as a defining interval. It has been hypothesized that a reduction in this interval could potentially lead to premature and rapidly propagating contractions in DES. A major point of focus has been nitric oxide (NO) due to its role in the inhibitory pathway of the myenteric plexus. NO-induced deficiency by administration of scavengers has been shown to induce simultaneous esophageal contractions, while NO repletion reversed it. Opioids, which have been associated with DES, increase NO secretion and inhibit neuronal excitation [1-2]. In one retrospective study, esophageal dysfunction like DES was more prevalent in those taking opioids for three months or longer [5]. Fortunately, these dysfunctions have been shown to resolve once the medication is stopped [1]. Psychotropic medications could also play a role, as almost half of DES patients use them per one demographic study [1,6]. Psychiatric diseases are also associated with chronic pain, which could lead to the overuse of opioids. 

Gastroesophageal reflux disease (GERD) might also be related to DES. Abnormal 24-hour pH monitoring is found in the majority of patients with GERD symptoms and DES, with some patients experiencing symptom improvement with anti-reflux treatment. It is believed that exposure to stomach acidity may modify the afferent nerves of the peristaltic peripheral pathways and thus lead to DES, however, this relationship is debatable. Interestingly, the abnormal pH monitoring was seen only with typical GERD symptoms, and not in those with chest pain or dysphagia [1,7]. 

Spastic achalasia is another disorder believed to be pathophysiologically related to DES. Achalasia is similarly classified as a disorder of deglutitive inhibition with dysfunction of the nitrogenic inhibitory pathway of esophageal smooth muscle, though in achalasia this inhibitory pathway is lost completely. Some cases have demonstrated DES progression to achalasia type 3 using HRM [1-2]. 

Presentation and diagnosis

The clinical presentation of DES is variable and its symptoms are episodic in nature. Most commonly, DES presents as esophageal dysphagia described as difficulty swallowing and/or a sensation of food getting stuck in the throat or chest. The uncoordinated contractions of the esophagus may lead to retrosternal non-cardiac chest pain requiring an initial rule out of acute coronary syndrome. Typical and atypical (e.g., asthma, coughing, hoarseness) GERD symptoms, weight loss, and vomiting are other attributable symptoms [1-2]. While the association of symptoms to feeding may be helpful, it is not required to diagnose DES [8-9].

Given the heterogeneity and non-specific character of its presentation, diagnosing DES requires specialized testing tools. Endoscopy is the modality used initially to exclude secondary motility disorders (eg, hiatal hernia, Schatzki’s ring, neoplasm) [10]. It also examines lower esophageal sphincter (LES) patency which helps in characterizing other diseases in a similar spectrum to DES, such as achalasia type 3 and GERD [2]. While endoscopy is not done to confirm DES, it may display behaviors suggestive of a motility disorder such as spastic, vigorous, and/or uncoordinated distal esophageal contractions with retained saliva or liquid in the esophageal lumen. These features, however, can be easily missed given the sporadic nature of DES [1].

**Figure 1:**
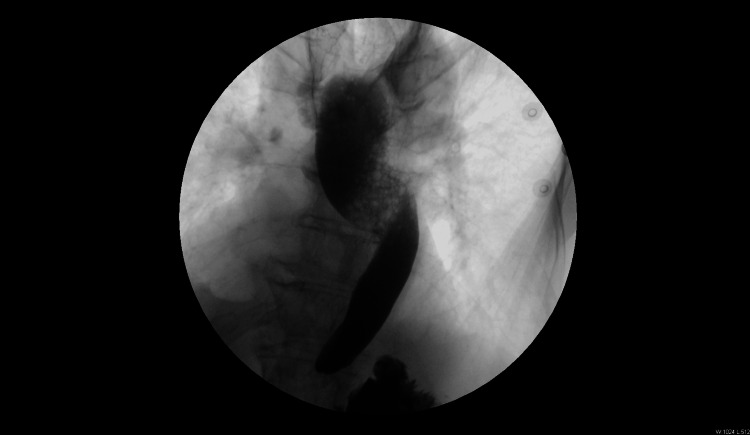
Barium swallow Barium esophagram showing the classic corkscrew pattern. Image taken from patient under care of the authors.

Barium esophagram is another adjunctive diagnostic method in dysphagia, though its sensitivity for motility disorders falls below 70%. The “corkscrew” or “rosary bead” appearance is a classic finding (Figure 1) [10]. 

The gold standard method for diagnosing DES is HRM, which is interpreted based on the Chicago Classification (CC) standardized system. According to the latest version (CC v4.0), DES is defined by premature contractions in at least 20% of swallows in the setting of normal LES relaxation in those with dysphagia or non-cardiac chest pain. This is an important distinction from the older CC which did not require relevant esophageal symptoms. Another addition to the CC v4.0 is the acknowledgment that intrabolus pressure on HRM can lead to the overdiagnosis of DES. Intrabolus pressure can be differentiated from contractile activity by its more homogenous appearance on Clouse plots and by adjusting the isobaric contour [9].

**Figure 2:**
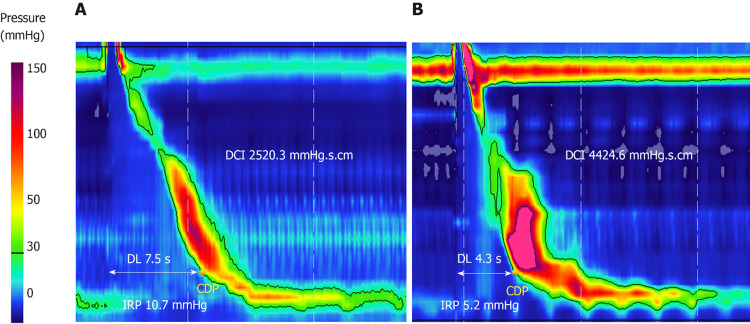
High-resolution manometry demonstrating a normal (A) and premature (B) swallow. Notice the short (< 4.5 s) DL and normal LES relaxation (IRP ≤ 15 mmHg) characteristic of DES. DL: Distal latency; CDP: Contractile deceleration point; IRP: Integrated relaxation pressure. Image by Harika Gorti et al. Licensed under CC BY-NC 4.0.  Source: [1]

Premature contractions are defined by a distal latency (DL) of < 4.5 seconds (Figure 2). DL is measured from the start of relaxation of the upper esophageal sphincter to the contractile deceleration point (CDP) of the peristaltic wave, normally 2-3 cm above the LES. CDP marks the transition from esophageal peristaltic clearance to esophageal emptying and is found where the slope of the pressure curve changes from positive to negative. Normal LES relaxation is defined by an integrated relaxation pressure (IRP) of ≤ 15 mmHg. Notably, DL can be falsely low in large hernias due to the shortening of the esophagus [9-10].

In order to increase both sensitivity and specificity in inconclusive cases, provocative maneuvers in the supine [multiple rapid swallows (MRS)] and upright [rapid drink challenge (RDC)] have been included in CC v4.0. The goal of MRS is to evaluate the peristaltic reserve of the esophagus by stimulating a rapid series of repetitive contractions, based on the hypothesis of progressively falling esophageal inhibition. RDC can detect esophagogastric junction (EGJ) obstruction and esophageal shortening that can be found in achalasia and other motility disorders [9-10].

Another important update in CC v4.0 was in regard to achalasia type 3. Per the previous CC, the difference between the two was based solely on IRP (normal in DES, elevated in achalasia). CC v4.0 now includes the absence of peristalsis as part of achalasia type 3 while acknowledging that occasional normal peristalsis can be seen in DES [9]. 

When it comes to GERD, it might be of benefit to perform pH monitoring in those with predominantly typical or atypical reflux symptoms and to repeat HRM after anti-reflux treatment, as manometric findings may improve [1-2,7,9,11]. 

**Figure 3:**
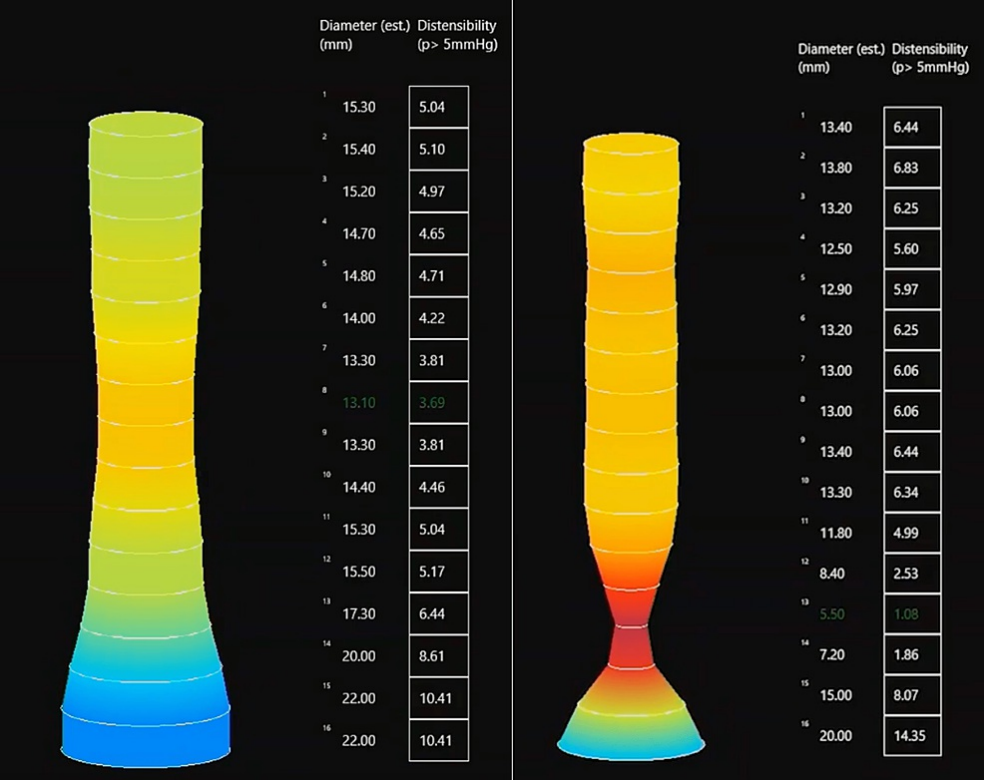
Functional lumen imaging probe Functional Lumen Imaging Probe (FLIP) of a patient with chronic dysphagia. Creation of the authors. We can see a normal (left) and low (right) distensibility index (DI) at the distal esophagus. A DI <2.0 mm2/mmHg and esophagogastric junction diameter of < 13 mm are characteristic of DES.

While HRM remains the gold standard, a newer modality called functional lumen imaging probe (FLIP) is being increasingly utilized as a complementary tool in evaluating esophageal motility (Figure 3). FLIP assesses esophageal luminal diameter and distensibility using impedance planimetry. Its catheter includes a distal pressure calculator and multiple impedance sensors surrounded by a distensible balloon. The catheter is inserted either nasally or orally down to the EGJ where it is inflated, generating a radial force against the esophageal wall. Data is analyzed in real-time and displayed as distensibility index (DI) and esophagogastric junction (EGJ) diameter. DI is the ratio of the EGJ cross-sectional area to intra-balloon pressure and is normally > 2.0 mm2/mmHg, while the EGJ diameter is > 13 mm. DI is often low in DES, indicating low compliance of the esophagus, while EGJ diameter tends to be normal or slightly reduced [10,12]. FLIP can also topographically display secondary peristalsis from balloon distension as anterograde or retrograde contractions in the esophageal body. Repetitive retrograde contractions or sustained occluding contractions secondary to balloon distension can be encountered in DES [9]. FLIP might better detect secondary peristalsis when compared to HRM, as its catheter is less contact-dependent and can detect non-lumen occluding contractions [13]. The data by FLIP can be objectively interpreted in real-time, is less operator dependent, and allows for a more complete picture of esophageal physiology as compared to HRM. It can also predict the clinical outcomes of myotomy, whether done surgically or endoscopically [14]. The data on the LES makes FLIP particularly useful in the evaluation of achalasia and esophagogastric junction outflow obstruction (EGJOO), as these may be associated with DES [1].

Management

Due to its unclear pathophysiology, treating DES is quite challenging. Both pharmacologic and more invasive approaches have been developed for management. The goal is to relieve symptoms rather than normalize manometric findings. 

Proton Pump Inhibitors (PPIs)

One of the clinical phenotypes of DES is reflux-like symptoms [9,15]. Reflux disease might also be present along with DES. 24-hr pH monitoring studies frequently show abnormal distal esophageal acid exposure in most esophageal hypomotility patients with typical reflux symptoms [7]. Therefore, a trial of PPI should be considered if there is a high likelihood of GERD. Likewise, pH testing while off PPI should be considered if there is a low likelihood of GERD.

Smooth Muscle Relaxants

Agents alleviating esophageal smooth muscle contraction are helpful in symptom relief. Nitrates and phosphodiesterase 5 inhibitors (PDE5i) increase the availability of NO, facilitating smooth muscle relaxation and transiently decreasing contractions. Long-acting nitrates such as isosorbide dinitrate are effective for non-cardiac chest pain and dysphagia if taken about 30 minutes before a meal. Calcium channel blockers (CCBs) induce smooth muscle relaxation by inhibiting L-type Ca channels [1]. Sublingual nifedipine is equally effective but relatively shorter in acting in comparison to nitrates. PDE5i, Sildenafil 50 mg in one clinical trial showed manometric improvement in nine out of 11 patients with esophageal motility disorder, with symptomatic improvement in only 4 [16]. Use of these agents is limited by their short duration of action requiring multiple dosing and frequent adverse effects such as headache, hypotension, and pedal edema.

Peppermint Oil

Peppermint oil relaxes gastrointestinal smooth muscle by reducing the influx of extracellular calcium ions [17]. Studies have shown both a reduction in peak current amplitude and an increase in the rate of current decay, indicating that the pharmacological activity of peppermint oil resembles that of dihydropyridine CCBs [18]. In a case series of eight DES patients who received peppermint oil, manometric studies showed the elimination of simultaneous esophageal contractions in all patients, and the number of multiphasic, spontaneous, and missed contractions also improved [19]. Another study by Khalf et al. showed symptomatic improvement in 24 out of 38 patients with esophageal motility disorder, where 83% response was seen in those with DES [2]. Peppermint oil is better tolerated in comparison to nitrates and CCBs and should be studied further for esophageal motility disorders. 

Overall, pharmacological therapy has only limited utility due to lack of efficacy, need for frequent dosing, adverse effect profile, and inadequate clinical trials. If pharmacologic management fails to resolve symptoms, a more invasive approach with endoscopy can be attempted. 

Botulinum Toxin Injection

Botulinum toxin, also called botox, is a potent neurotoxin that causes reversible chemical denervation and partial paralysis. This is done by inhibiting the presynaptic release of acetylcholine in muscle fibers [20]. Injection of botox into the distal esophageal sphincter is a well-studied and effective short-term treatment for achalasia, especially in medically high-risk patients [21]. Evidence supporting the use of botulinum toxin injection as a treatment for non-achalasia esophageal motility disorders like DES is inconsistent. However, if botulinum toxin is used for this indication, dysphagia is probably the target symptom and there is data to advocate injection into the distal esophagus in conjunction with botulinum toxin injection into the LES [22]. Hoeij et al. reported mild complications (clavien-dindo grade I) in 48 out of 386 patients who received botulinum toxin injection for the treatment of esophageal motility disorders (total of 661 procedures and 30% of patients with DES) and one fatal incident from acute mediastinitis, although DES itself was not a risk factor for complications from botox injection [23]. 

Peroral Endoscopic Myotomy (POEM)

POEM is a novel intervention for the treatment of achalasia and other esophageal motility disorders. Although it has largely been studied for achalasia, POEM has been in recent years explored as a treatment modality for non-achalasia spastic esophageal motility disorders, including DES. A meta-analysis of five studies reporting outcomes of POEM in non-achalasia esophageal disorders, including DES, demonstrated clinical success (Eckardt score ≤3 or <4) extending beyond 60 months [24]. On the contrary, other case reports and case series showing the efficacy of POEM for non-achalasia esophageal motility disorders have either unknown or short-term follow-up and risks of publication bias [25]. 

Considering the benign course of primary esophageal motility disorders like DES and the lack of high-quality studies on the efficacy and safety of invasive procedures like POEM, the European Society of Gastrointestinal Endoscopy advises caution in treating such conditions using POEM. It should be considered when medical or less invasive management fails and the patient is experiencing severe dysphagia causing weight loss. Another drawback of POEM is the lack of widespread availability of instrumentation and expertise. Future high-quality randomized-controlled trials are required to evaluate the efficacy and safety of POEM in primary esophageal motility disorders, including DES.

Surgery

Surgery is usually only considered for refractory and severe disease since the prognosis of DES seems favorable, even without treatment [10]. A small prospective study of 20 patients by Leconte et al. showed that extended myotomy with anterior fundoplication for severe DES led to improvement in chest pain and dysphagia scores for over a 50-month follow-up, with post-myotomy GERD in 2 patients [26]. Future high-quality randomized-controlled trials are required to compare the safety and efficacy of POEM versus myotomy for DES.

Prognosis

The course of DES appears to be benign without an increased risk for mortality or carcinoma development [10]. The disease however did show a potential to progress to achalasia type 3 and esophageal diverticula [1-2,26].

## Conclusions

DES is a rare motility disorder characterized by premature contractions of the distal esophageal smooth muscle. The pathophysiology likely involves an imbalance between the inhibitory and excitatory pathways of the smooth muscles. Diagnosing DES is challenging due to its variable and episodic symptoms. HRM is currently the gold standard for diagnosis and is interpreted using the CC. Recent updates to the CC have improved the accuracy of the diagnosis, including the incorporation of provocative maneuvers and the differentiation of DES from achalasia type 3. FLIP is a newer modality that provides complementary information to HRM, allowing for a more comprehensive evaluation of esophageal motility. Managing DES is difficult due to the limited understanding of its pathophysiology. Treatment options include proton pump inhibitors for concurrent GERD symptoms, smooth muscle relaxants such as nitrates and calcium channel blockers, and peppermint oil. However, pharmacological therapy has limited efficacy and frequent dosing requirements. Invasive approaches such as botulinum toxin injection and POEM may be considered once more conservative measures fail. Further multi-center randomized clinical trials will help in asserting the role of POEM in treatment. DES remains a challenging condition to diagnose and treat, but advancements in diagnostic techniques such as HRM and FLIP have improved our understanding of the disorder. Further research is needed to elucidate the underlying mechanisms of DES and to develop more effective treatment strategies to alleviate symptoms and improve the quality of life for affected individuals.
